# Reviews

**Published:** 1881-04

**Authors:** 


### Reviews.

---

**A Practical Treatise on the Medical and Surgical Uses of Electricity, etc.** By GEO. M. BEARD, M. D., and A. D. ROCKWELL, L.L. D. 3d edition. With nearly two hundred illustrations. New York: William Wood & Co. 1881.

This book is too well known to the profession as the largest and most perfect work on electricity to require any recommendation from us. Several additions have been made, and a few new chapters added, by which the usefulness of the work has been increased.

---

**A Manual of Medical Jurisprudence.** By ALFRED SWAINE TAYLOR, M. D., F. R. S., Fellow Royal Coll. of Physicians, etc. Eighth American edition from the 10th London edition containing the author's latest notes, made expressly for this edition. Edited by John I. Reese, M D., Prof. Medical Jurisprudence and Toxicology in the University of Pennsylvania. With illustrations. Philadelphia: Henry C. Leas, Sons & Co. 1880.

Taylor's Medical Jurisprudence is so well known to the members of the medical and legal profession that it is unnecessary to say anything commendatory of the work. The additions to this volume are, however, quite numerous, and bring the work up to the level of the latest results of the experience and research of the lamented author. The medico-legal student is to be congratulated that the master hand was spared to complete the revision of this great work. It will long remain the most reliable manual of medical jurisprudence in the English language, and should find a place in the office of every physician and lawyer.

---

**Atlas of Skin Diseases.** By LOUIS A. DUHRING, M. D., Professor of Skin Diseases in the Hospital of the University of Pennsylvania, &c. Part viii. Philadelphia: J. B. Lippincott & Co. 1880.

The present number gives accurate illustrations of Erythema Multiforme, Psoriasis. Syphiloderma (Tuberculosum) Tinia Trychophytina (Circinata et Tonsurans), and the text, as in preceding numbers, contains a clinical lecture on the disease illustrated on the plate. The author treats the subject in a practical manner, and furnishes a valuable aid in the diagnosis of cutaneous diseases, as well as practical suggestions as to their treatment. This number compares favorably with those that have preceded it, and fully maintains the reputation of the publishers in the assurances given when undertaking the work of presenting to the profession a guide and a help in unravelling the intricacies of this department of medicine. We endorse the work and heartily recommend it.

---

**Photographic Illustrations of Cutaneous Syphilis.** By GEORGE HENRY FOX, A. M., M. D., Clinical Lecturer on Diseases of the Skin. College of Physicians and Surgeons, New York. Forty-eight plates from life, colored by hand. New York: E. B. Treat, No. 757 Broadway.

We have received Nos. 4, 5 and 6 of this work, the previous numbers having been noticed in the December number of the JOURNAL. The endorsement then given to the initial numbers are deservedly due to those recently received. No. 4 contains plates illustrating syph. papulosum et pustulosum, syph. pustulosum, syph. pustulosum corymbiforme, onchia syphilitica; No.

5 contains plates illustrating syph. papulosum humidum, syph. papulo-squamosum, hydroa pemphigus iris; No. 6 contains plates illustrating eczema squamosum, syph. squamosum circinatus, syph. tuberculosum ulcerativum, syph. squamosum gyratum, circenatum, syph. tuberculosum.

It will be seen that the varieties of syphiloderma here illustrated are quite extensive and well selected as far as the practical benefit to the profession, for which the work is published, is concerned. If the succeeding numbers are as judiciously arranged, the work cannot fail to be of great service in this important department of medicine. We are assured that in endeavoring to meet a want long felt by every physician, the author is doing an essential service to humanity.

---

**Syphilis and Marriage.** Lecture delivered at the St. Louis Hospital, Paris. By ALFRED FOURNIER, Professor a la Faculti de Médicine de Paris, &c., &c. Translated by P. Albert Morrow, M. D., Physician to the Skin and Venereal Department New York Dispensary. New York: D. Appleton & Co., 1, 3 and 5 Bond street. 1881.

This volume deals with a problem on the social life of the race, the most important with which the medical profession have to contend. We know of no other work upon this subject in the English language, and we congratulate the translator and the publishers in offering this, the most reliable authority extant, on the special subject of which it treats. We are sure the profession will welcome this work, inasmuch as questions frequently arise in which authority is required to confirm settled convictions, upon this and kindred questions. In examining the work we find it grows in interest as we read, and the importance of the subject in its relations to the fruits of the marriage state, cannot be over-estimated.

The author treats the question of heredity, both natural and potential, the condition of the admissibility to marriage to the syphilitic, the importance of specific treatment as a safeguard against risks, the immunity acquired through the lapse of time, and other qualities which follow in the discussion of this vital subject. In the notes in which are given clinical experience to substantiate his views, he furnishes abundant evidence to satisfy the professional reader that the position taken upon the subject under consideration is incontrovertible. We think the translator has performed the labor assigned him with great fidelity. The volume should be in the library of every physician residing in our large centres of population.

---

**Health Premiers—The Heart and its Functions.** New York: D. Appleton & Co., 1, 3 and 5 Bond street. 1881.

This work constitutes the eighth of this series, and aims to popularize the essential facts and principles connected with the circulatory system. We think the profession will find such a work to be of essential service in imparting information to their patients.

---

**Diagnosis and Treatment of Ear Diseases.** By Albert H. Buck, M. D. William Wood & Co.

The small space allotted to the notice of so excellent a book as this is entirely inadequate to give an idea of its worth. If the others in this series published by Wood, are to be regarded as concise and fair expositions of the subjects treated, this one must have a foremost rank among them. The writer opens to us a panorama of wood pictures of the various diseases of the ear, as they appear in every-day practice, and to make a complete whole, has introduced occasional illustrations from the experience of other practitioners.

After giving a sketch of the anatomy and physiology of the organ, he shows how the examination of patients is to be conducted, and then takes up in detail the different diseases, relating to the auricle, the canal, the tympanic cavity, etc. In the arrangement of these more regard is had for their clinical features than for pathological distinctions. For instance, the diseases of the middle ear are divided in general into the purulent and non-purulent forms. A classification which some pathologists might object to, saying that they are often but different stages of the same process, and yet for the ordinary practitioner such an arrangement is very convenient. This is only one example of many which might be cited to show the eminently practical character of the book, which renders it, as a whole, one of the best manuals on the subject available to English readers.

---

**A Treatise on the Materia Medica and Therapeutics of the Skin.** By HENRY G. PIFFARD, A. M., M. D., Professor of Dermatology University of the City of New York. New York: Wm. Wood & Co., 27 Great Jones st. 1881.

The publishers have evidently designed in their selection of subjects and authors for the present series to supply the most urgent needs of the profession, and carrying out this purpose, we endorse heartily, not only, the aim and object of the present work, but also the eminent fitness of Professor Piffard to carry the plan in a successful end. The author has been an able contributor to the medical press for several years. The products of his pen have been received with increasing favor and confidence by the profession. He has the independence to advocate principles in therapeutics and methods in pharmacy, which have not descended to the present generation through the regular orthodox line, but which are nevertheless founded in a sound, rational and practical basis. Several years ago the author read a paper before the New York Academy of Medicine on the comparison of milk sugar triturations of mercury and its salts with the officinal preparations of this drug. We see that in this work he emphasizes the greater efficacy of the triturations. The same independence is noticeable in other medicines which he proposes for use, and it would be well for the profession to give consideration to the views thus presented, inasmuch as they are substantiated by his large clinical experience. He aims especially to impart correct knowledge of the drugs that affect the skin and the manner of their action. In view of the increasing attention now given to the study of dermatology through the works of Fox and Duhring, the publication of this excellent work, which forms one of Wood's library of standard medical authors, is most opportune and will be fully appreciated by the profession. No other work in this entire series compares with the present in real practical value and usefulness. As such we cordially recommend it.

---

**An Elementary Treatise on Practical Chemistry and Qualitative Inorganic Analysis.** Specially adapted for use in Laboratories of Schools and Colleges and by beginners. By FRANK CLOWES, D. Sc , Fellow of the Chemical Society of London and Berlin, etc. With illustrations. From the second English edition. Philadelphia: Henry C Lea.

This work is deservedly a favorite with many teachers as a hand book for the student in the laboratory. It is well written and arranged, unincumbered with theoretical deductions, and the analytical methods given are those which are the most simple and easy of execution. It is especially well adapted to furnish a course of instruction in practical chemistry in our public and other schools.

---

**A Manual of Diseases of the Throat and Nose.** By FRANCKE H BOSWORTH, A. M , M. D., etc. New York: William Wood & Co. 1881.

The members of the Laryngological Association are, it seems, at present possessed with a mania for writing books on diseases of nose, throat, catarrh, etc. Quite a goodly number of manuals and monographs on these subjects have appeared of late, and it seems to us the field is rather overworked just at present. Necessarily, therefore, these works offer in a great measure very little, which may be said to be new, while they all contain the same chapters about examinations and methods of treatment, with almost the same pictures. As a record of the personal experience of the author, this work is of interest, and to be recommended to the profession. He tells honestly when he was successful and when he failed in the treatment of these affections. The classification of diseases is sensible and brief, and does away with many of the misleading names with which the larger books are filled. Altogether the book is written in a clear and forcible style, and tastefully gotten up, and it will occupy a worthy place among the recent publications on this subject.

---

**Chemical Physiology and Pathology.** With lectures upon Normal and Abnormal Urine. By VICTOR C. VAUGHAN, M. D., Ph. D., Lecturer on Medical Chemistry in the University, of Michigan. 3d edition, revised and enlarged. Ann Arbor: Ann Arbor Printing and Publishing Co. 1880.

When the first edition of this work was issued, we called attention to its value, and commended it to the attention of all physicians. That the work has already reached a third edition is conclusive evidence of the correctness of the opinion then expressed. This edition is materially improved and enlarged, and its value and convenience enhanced by the addition to the volume of the plates, illustrating most of the crystaline amorphous and organized deposits found in the urine.

---

**John Hunter and his Pupils.** By PROF. GROSS. Philadelphia: Presley Blakiston, Publisher. 106 pages. Price $1.50.

The exceedingly attractive dress in which this work is presented to the public, is by no means its chief recommendation, though we cannot but speak in high terms of this somewhat superficial quality. The paper, type, and above all, the fine photograph of a spirited portait of Dr. Hunter, by Sir Joshua Reynolds, would invite the readers to even dull pages, while the agreeable first impression is deepened by further acquaintance with the author's pleasing style. He has, however, a higher object in view, as a result of his efforts, than catering to the popular eye or casual reader, viz.: that of giving to the American student a succinct account of the life and services of one whom he regarded as the Newton of the medical profession, thereby inducing emulation of his example, and a more extensive study of his writings.

Perhaps Prof. Gross' unbounded admiration for Dr. Hunter may cause him to paint in rather too glowing colors his undoubtedly remarkable character; serious faults are condoned or glossed over, but the strong points are admirably brought forward and cleverly demonstrated by the pithy remarks of the great surgeon. Owing to defects in early education, Hunter himself did not wield an easy pen, yet he was a strenous advocate of its use. Putting one's thought into writing "resembles," he said, "a tradesman taking stock, without which he never knows what he possesses or in what he is difficient." Were this advice more heeded by physicians generally, not only would it tend to their own improvement, but many a valuable clinical experience would not, as now, be lost to the profession.

The latter part of the book is devoted to brief sketches of some of Hunter's distinguished pupils, among them Jenner, Aternathy, Physick and Sir Astley Cooper. Short accounts also, of a few English contemporaries, impart additional interest to the book, and render it a valuable acquisition to the student's as well as the physician's shelves.

---

**The Compend of Anatomy,** for Use in the Dissecting Room and preparing for Examinations. By JOHN B. ROBERTS, M. D. Philadelphia: C. C. Roberts & Co., 1118 Arch street. 1881.

It is a convenient little anatomy, and will serve its purpose in the dissecting room.

---

**How Persons Afflicted with Bright's Disease ought to Live.** By JOSEPH F. EDWARDS, M. D. Philadelphia: Presley Blakiston, 1012 Walnut street. 1880.

A little treatise of about 80 pages, 12 mo. In the words of the author, all that he says amounts to nothing more or less than the injunction to live *reasonably* and *moderately;* and we have nothing to add.

